# DNA fingerprinting reveals varietal composition of Vietnamese cassava germplasm (*Manihot esculenta* Crantz) from farmers’ field and genebank collections

**DOI:** 10.1007/s11103-021-01124-0

**Published:** 2021-02-25

**Authors:** John Ocampo, Tatiana Ovalle, Ricardo Labarta, Dung Phuong Le, Stefan de Haan, Nguyen Anh Vu, Le Quy Kha, Luis A. Becerra Lopez-Lavalle

**Affiliations:** 1 International Center for Tropical Agriculture (CIAT), Cali, Colombia; 2 National University of Colombia (UNAL), Palmira, Colombia; 3 Agricultural Genetics Institute (AGI), Hanoi, Vietnam; 4 Institute of Agricultural Sciences for Southern Vietnam (IAS), Ho Chi Minh, Vietnam

**Keywords:** SNP markers, Cassava varieties, Germplasm, Genetic variability

## Abstract

**Key message:**

A molecular analysis using informative SNP markers in 1570 clones of cassava from Vietnam reveals varietal composition from farmers’ field and genebank collections

**Abstract:**

Cassava is the most important smallholder cash crops in Southeast Asia and is especially used in industrial products. Yet, systematic genetic studies on molecular markers from Vietnamese germplasm have not been considered for breeding and conservation programs. We conducted a molecular analysis of 1570 clones of cassava germplasm from farms across six agro-ecological zones using informative SNP markers. We unraveled the genetic diversity and population structure and provided insights into the value of breeding and conservation programs. Duplicated genotypes comprised 98% of the total sample of the Central Highlands region. Ninety-six SNPs were amplified Central Highlands and South East provinces had the highest allelic richness, covering up to 83% of alleles. The average observed heterozygosity (*Ho* = 0.43) was slightly higher than expected (*He* = 0.40) across SNP markers, suggesting an excess of heterozygotes plants. Diversity indexes indicated that cassava populations from North West and Eastern Vietnam are genetically diverse (mean *He* = 0.40). Genetic parentage tests identified 85 unique genetic groups within the varieties KM94, KM419, BRA1305, KM101, KM140, PER262, KM60, KM57 and two unidentified varieties, which accounted for 82% of the frequency distribution. KM94 is the most dominant variety in Vietnamese farms surveyed (38%), reflecting its superior quality and productivity. Discriminant analysis of principal components (DAPC) revealed four main subgroups, which were partially corroborated by neighbor joining (NJ) analyses. After removing duplicates, 31 unique genotypes were distributed across five of the agro-ecological zones. These were well distributed in the subgroups revealed via DAPC and NJ analyses. The genetic groups identified herein could be used to select unique accessions that should ideally conform with ex situ germplasm collections and identify areas where on-farm conservation programs should be targeted. Newly identified genotypes may also contribute as genetic breeding resources that could be used to adapt cassava to future changes and farmers’ needs.

**Supplementary Information:**

The online version contains supplementary material available at 10.1007/s11103-021-01124-0.

## Introduction

Cassava, also termed as yuca, manioc, or *sắn,* in Vietnamese, (*Manihot esculenta* Crantz), is a vegetatively propagated crop that was domesticated in South America approximately 8500–7000 years ago (Allem et al. 1994; Olsen et al. 1999). Cassava is an outcrossing species, even though natural self-pollination may also occur (Ramos et al. 2019). The species has 2*n* = 36 chromosomes (Carvalho and Guerra 2002) and a genome size of 746 Mb (Wang et al. 2014). It is one of the world’s most important stable tropical crops, with annual global production of 270 million tons. This crop is the primary staple food for > 800 million people globally and is the third most important crop in the tropics after rice and maize (Lebot 2008; Visser et al. 2010; FAOSTAT 2018). Starchy cassava roots have various uses, including human consumption, starch production, and animal nutrition (Debouck et al. 2011). Cassava crops are typically produced by smallholder farmers, often under marginal conditions in humid and semi-humid tropical areas. Hence, cassava is adapted to a wide range of environments and is tolerant to drought and acidic soils (Visser et al. 2010). Crops are produced well under low-external input conditions but are known to respond well to supplements, such as fertilizer and manure. Cassava seed systems are typically farmer-managed and are dependent on informal exchange networks (Delaquis et al. 2018).

The main global collection of cassava germplasm is maintained at the Future Seeds genebank of the International Center for Tropical Agriculture (CIAT) in Colombia (Visser et al. 2010). The CIAT conserves this publicly accessible germplasm of 6592 accessions from 28 countries to assure future availability of diversity and genetically enhanced crops that perform optimally in terms of yield stability, nutrition contents, and climate change adaptation (Debouck et al. 2011). The existing genetic diversity among these germplasm follows natural selection over millennia and also farmer selection enhancement through breeding programs (Hershey and Debouck 2010; Ceballos and Hershey 2017). In Asia, expanding industrial demands for starch and ethanol have triggered the successful development of new hybrids.

Traders introduced cassava into Africa in the 1500s and into Asia in the 1800s, first to the Philippines, India, and Indonesia and later to Malaysia, Thailand, Vietnam, and China (Kawano 1978; Byrne 1984; Malik et al. 2020). Initially, cassava was grown mainly as a food crop but was soon used for small-scale starch processing and on-farm pig feeding. In Asia, bitter cassava varieties are typically used for the production of starch and the local sweet varieties are grown for human consumption (Lamprecht 2015; Kim et al. 2015). Asia is now the largest global cassava trader, with 31% of global production in Asian countries. Among these, Vietnam has become one of the major cassava producers of the region, with crops representing an export value of well over US$1.5 billion (Agency of Foreign Trade 2018). Over 70% of Vietnam’s fresh root production is destined for export, mainly to China, South Korea, and Malaysia (Aye et al. 2015). In 2015, the total cassava starch production in Vietnam was approximately 560,000 tons (FAOSTAT 2018). The average cassava cropping area per farm for all of Vietnam is 1.8 ha, with extremes of 4.0 and 0.7 ha for the Southeast and the North Mountainous Regions, respectively.

Research on cassava has made remarkable progress since 1988, when Vietnamese institutions, such as the Institute of Agricultural Sciences for Southern Vietnam (IAS) and the Vietnam Cassava Research and Extension Network (VNCP), began cooperating with CIAT (Kim et al. 2015; Malik et al. 2020). Subsequently, national production increased from 1.9 million tons in 2000 to 11 million tons in 2015 (Agency of Foreign Trade 2018; Malik et al. 2020). This increase followed an area expansion from 278,000 to 560,000 ha and marked increases in yield/area from 9 t/ha in 1993 to 19 t/ha in 2015 (Kim et al. 2015; Malik et al. 2020). Crucially for yield increases, the new high-yield and high-starch cassava varieties KM60, KM94, KM95, KM95-3, KM98, KM98-1, KM98-5, KM98-7, KM140, KM419, and SM937-26 were introduced. Prior to the 1990s, improved varieties were developed using Rayong 1 from Thailand, which is a bitter cassava variety with high yields and starch contents (Kawano 2003). As a result, 19 new varieties were registered in the last 20 years (Malik et al. 2020). Currently, KM94 and KM419 varieties have been adopted by most farmers in Vietnam, and represent approximately two-thirds of all cassava grown in the country for processing (Aye et al. 2015; Le et al. 2019); however as much as 29% is consumed fresh representing an important source of dietary energy (Le et al. 2019). The intensification of production practices through the use of fertilizer, intercropping, and erosion and weed control have also contributed to the overall performance of cassava.

Cassava farming is a relatively recent practice in Vietnam, and the numbers of generations are insufficient for mutations and natural selection processes to have played major roles in diversification. Moreover, cassava landraces from Vietnam are conserved ex situ by both national and international agricultural institutes, and as part of IAS and CIAT the Hung Loc Agricultural Research Center (HLARC) presides over 20 national and 50 international accessions (Hershey and Debouck 2010). The local sweet varieties are grown over small areas and are typically used for local human consumption or animal feed (Kim et al. 2015; Lamprecht 2015). Cassava germplasm have been frequently characterized to determine their origins (Olsen 1999) and to investigate genetic diversity using molecular markers such as simple sequence repeat (SSR) markers (Fregene et al. 2003; Siqueira et al. 2009; Montero et al. 2011; Wangsomnuk et al. 2013; Fu et al. 2014; Lamprecht 2015). As alternative markers, single nucleotide polymorphisms (SNPs) are biallelic, locus specific, and co-dominant and are more abundant than SSRs. The related genotyping methods are also easy to automate and share among laboratories (Rafalski 2002; Padi et al. 2015; Singh et al. 2019). The first study to identify and characterize African cassava germplasm was performed by Kawuki et al. (2009) using a set of 26 SNPs markers. Ferguson et al. (2012) subsequently performed a more comprehensive analysis of the genetic diversity of cassava based on SNPs, and revealed greater diversity in germplasm from the Americas than in those from Africa. Brazilian cassava germplasm was also genotyped using SNP markers by Oliveira et al. (2014), who indicated the presence of a complex genetic structure with low associations between genetic diversity and geographic origin. Recently, a new set of cassava SNPs markers were developed at the International Center for Tropical Agriculture (CIAT) cassava genetics laboratory using next-generation sequencing information. This set of SNPs are composed of 96 markers highly informative and distributed in all 18 chromosomes of cassava. Thus, several studies in cassava have showed the utility of this markers, such as varietal identification (Floro et al. 2017), genetic variability (Peña-Vanegas et al. 2014) and breeding programmes (Becerra López-Lavalle 2017), allowing it to confirm genetics duplicates samples and to verify known pedigrees. As in previous studies, these investigations revealed high genetic diversity and a strong genetic structure in cassava germplasm. These authors also report high genetic differentiation of the traditional varieties of cassava among five ethnic groups from the Colombian Amazon. A similar study on DNA-based cassava varietal identification, using SNPs along with socioeconomic data analysis, has also been deployed to estimate the average adoption rates of improved varieties in Nigeria (Wossen et al. 2017, 2018).

In Vietnam, cassava germplasm have not been characterized in great detail. In particular, varietals have not yet been identified using DNA fingerprinting analysis. In a seminal study of genetic diversity among Vietnamese cassava varieties, morphological and SSR analyses distinguished high-yield varieties among 19 varieties (Nguyen et al. 2013). Similar studies were based on DNA polymorphisms of the *GPSS1* gene (Nguyen et al. 2015a, b), and SSR analyses (Nguyen et al. 2015a, b) showed high genetic diversity among 44 Vietnamese cassava varieties. In another study, genetic variations of cassava in 15 farms from North and Central Vietnam were identified using SSR markers, and local and improved varieties were distinguished (Lamprecht 2015). Recently, a study of the major cassava cultivars in Vietnam (KM94, SM 937-26, KM98-5, KM98-7, KM140, KM149, and XVP) offered partial morphological characterization of these varietals (Ha et al. 2016). Yet, no previous studies have systematically sampled the entire country or used SNP markers. Therefore, the specific objectives of this research were (1) to determine the overall genetic variability and population structure of cassava in Vietnam, (2) to infer the genetic diversity of cassava across the agro-ecological zones of Vietnam that provide insights into spatial distribution patterns, and (3) to compare on-farm diversity with accessions from selected genebank collections and make recommendations for future conservation.

## Materials and methods

### Study area and sample collection

The study uses a nationally representative sample of cassava farmers, following a multi-stage sampling design. We targeted the cassava producing areas that represents 95% of the total cassava production in Vietnam; covers 32 of the 64 provinces in Vietnam. Using power calculations and existing information from previous agricultural surveys, we estimated that the minimum sample size of the study should include at least 932 households. Then applying a probability proportional to size sampling method, we randomly select primary sampling units (villages) in the first step. In a second step, we randomly selected 12 households in each selected village. Villages located in areas with higher cassava production were more likely to be selected for the study. For selecting households for the study, we assigned equal probability of being selected to the list of households made available for each selected village. The final study consisted in 949 households in 79 villages across the country.

**Fig. 1:**
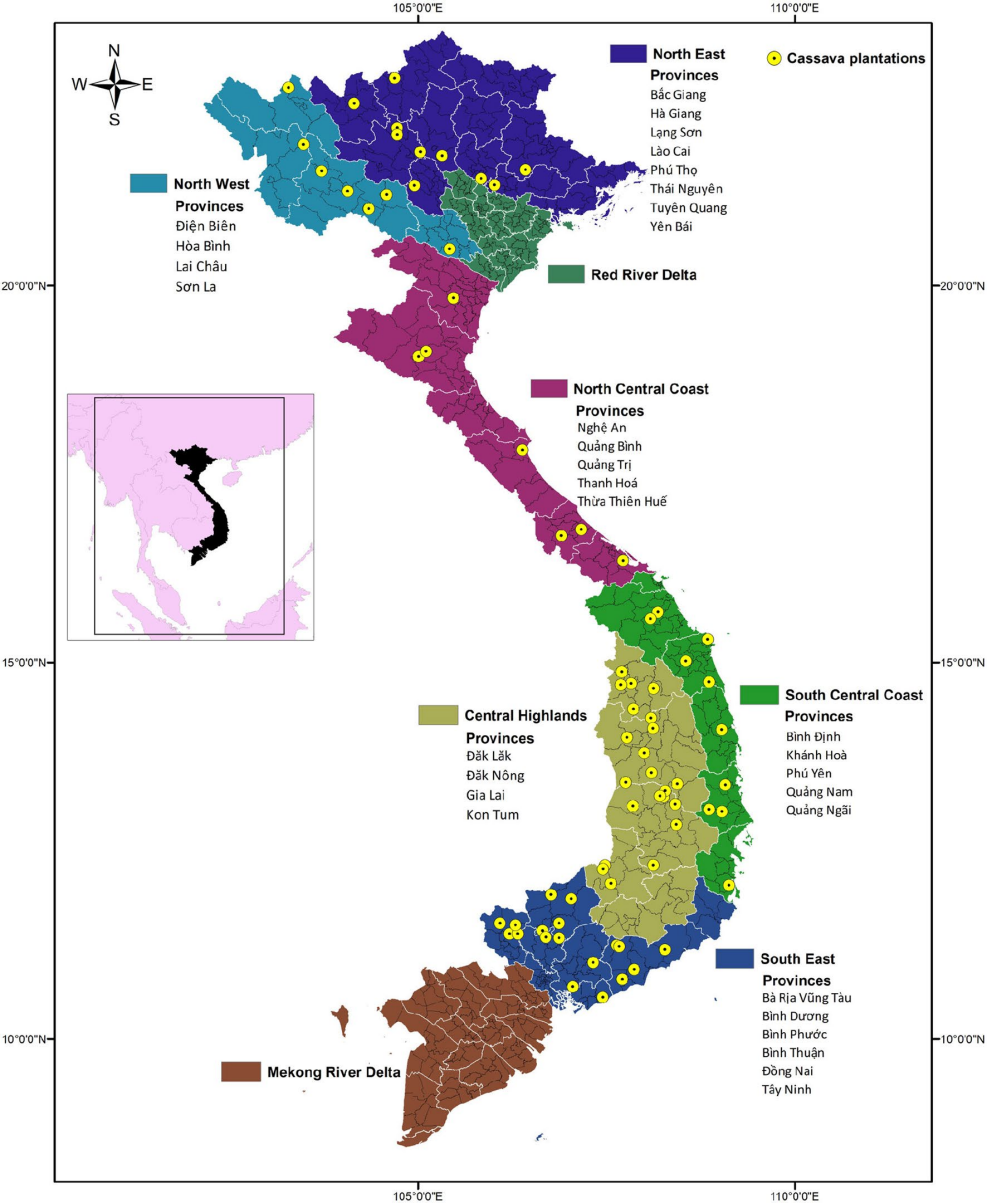
Dot map distribution of cassava plantations across different provinces and agro-ecological zones in Vietnam

Each selected household responded a survey question about the cassava production and commercialization. Additionally, each household were asked to provide a cassava sample for each of the cassava varieties reported by the household. The data and sample collection was conducted between October and December 2015. Each collection site was geo referenced using a GPS device and the local variety names were recorded from farmers. A total of 1570 cassava samples were collected in major cassava planting areas across Vietnam (Fig. 1). Samples were collected as stakes (singe plants, > 3 cuttings) and were tagged for ease of identification. The entire collection was planted in 2016 in a variety garden at the Root Crop Research and Development Center (RCRDC) of the Field Crop Research Institute in Hanoi, Vietnam.

### Genotyping of SNPs

Leaf samples were collected from the variety garden and DNA was extracted from each of the 1570 samples at the laboratory of the Agricultural Genetics Institute (AGI) in Hanoi, essentially using the CTAB-based DNA extraction protocol described by Doyle and Doyle (1990). Genotyping was then performed using a SNP-chip for Nanofluidic Dynamic Arrays (SNPY-Array; Fluidigm®, USA), as developed by the CIAT. This chip contains 96 SNPs and has been used previously to identify varieties and assess diversity in several studies (Peña-Venegas et al. 2014; Floro et al. 2017).

### Data analysis

#### Variety and duplicate identification

**Fig. 2:**
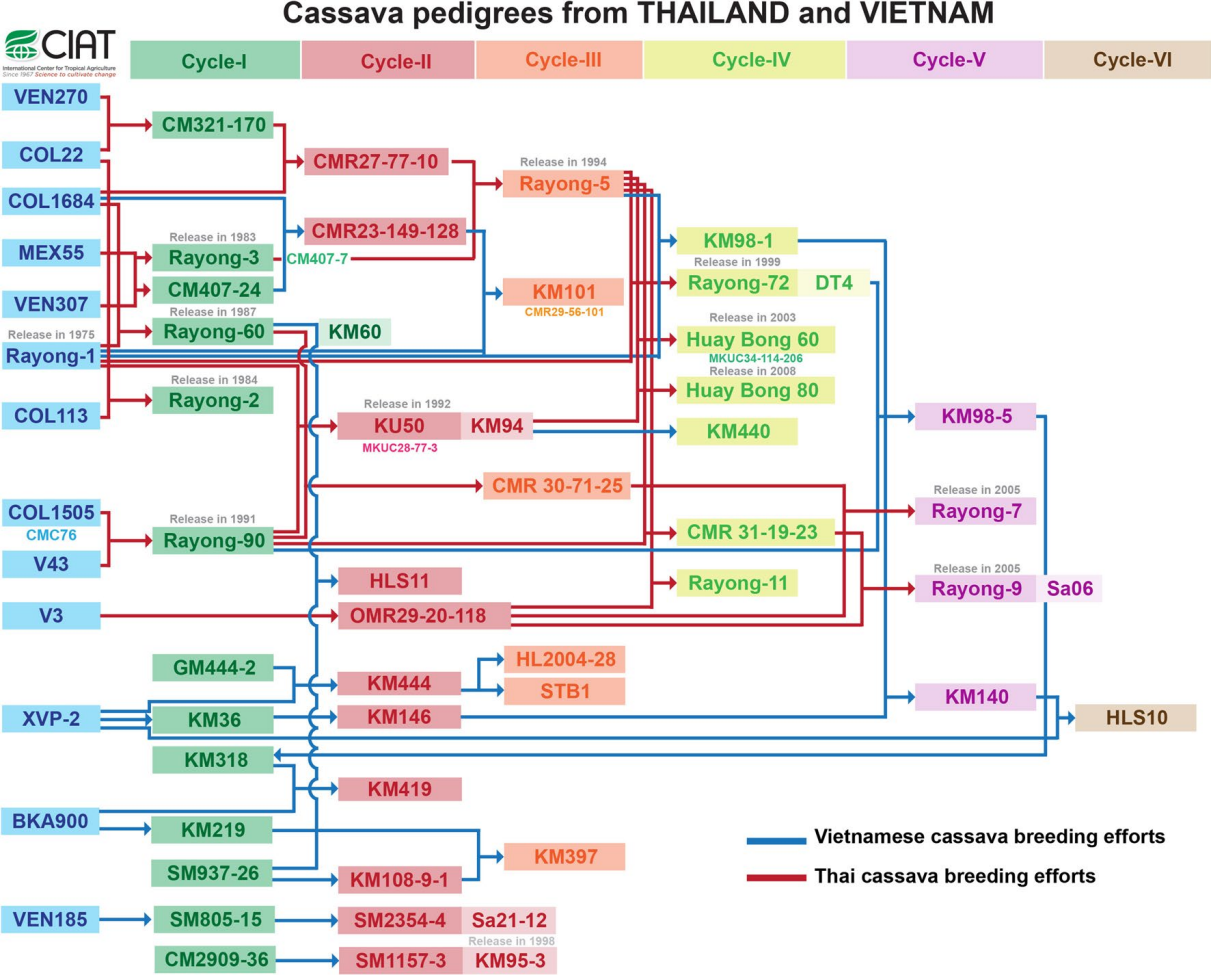
Pedigree of cassava varieties released in Thailand and Vietnam

Samples were compared with the single nucleotide variation library at the CIAT. This library is based on 2000 diverse genotypes, and comprises materials from ex situ collections that were assembled in national programs in Latin America (1600 genotypes; held at CIAT) and Asia (400 genotypes were provided by HLARC, RCRDC, and AGI). A comparative analysis was also performed using a genetic duplicate test based on the homozygous/heterozygous allele-call correspondence differences (< 3%) implemented in the next-generation sequencing experience platform (NGSEP; Duitama et al. 2014). Plants with identical allele calls at the same locus (across all 96 loci) were considered as single genotypes or duplicates. Following duplicates tests, we analyzed varietal relationships using the Kinship coefficient and determined 1st, 2nd, and 3rd degree relationship inferences among samples to reconstruct pedigrees where possible based on the strategy described by Fernandez et al. (2013) and Zhou et al. (2015); the kinship was calculated using a mathematical algorithm implemented in KING (Manichaikul et al. 2010). These analyses were complemented with previous information of cassava breeding pedigrees from Thailand and Vietnam (Fig. 2).

#### Genetic diversity

Parameters of genetic diversity were calculated using a set of samples that represented genetically differentiated genotypes within the six major agro-ecological zones of Vietnam. Average heterozygosity (*Ho*), gene diversity (expected heterozygosity, *He*), and polymorphism information contents (PIC) were determined using the diversity tool of the NGSEP platform (Duitama et al. 2014).

#### Population structure and cluster analyses

To determine genotype by genotype associations, discriminant analysis of principal components (DAPC) was performed using the Adegenet library that was developed for R. We used Bayesian information criteria to define numbers of subgroups for this dataset. We generated a genetic distance matrix using Power Marker V. 3.25 and used the “Shared allele” method to estimate genetic distances. Clustering was performed using the neighbor joining (NJ) method with MEGA v. 6 software, and was visualized and edited using the interactive tree of life (iTOL; https://itol.embl.de/) platform.

#### Frequencies of cassava varieties across Vietnam

Based on the genotype data generated using the 1570 samples collected across Vietnam, genotype frequencies were analyzed using geo referenced information. The genotype frequencies data matrix was plotted using the Geographic Information System ArcGIS Desktop Help 10.2 Geostatistical Analyst (http://resources.arcgis.com/en/help/main/10.2/index.html).

#### Spatial distributions of alleles

We generated a dot map of the cassava farms, from which samples were collected. Additionally, all alleles per locus were geo referenced for spatial distribution as described by van Zonneveld et al. (2012). A grid for allelic richness parameters was generated using the software DIVA-GIS 7.5 with a cell size grid of 0.05 min, which corresponds with approximately 0.1 km in the study area. Finally, we applied circular neighborhoods with a diameter of 0.5 degrees (approximately 55.5 km).

## Results

### Identification of cassava varieties and distribution by farmers

Farmers assigned a total of 97 names to the 1570 varieties that were collected in their fields, and only 3% of these were not given local names. Among named varieties, 33% were reported by a single farmer only, including Bac Quang, Bun Gong, Dong Nai, HL-S10, Indian Red, Short Kuc 94, and SM937-26. Some varieties were assigned the same names by farmers in at least four of the six agro-ecological zones. These included white, Green, High Yielding, and KM94.

**Fig. 3:**
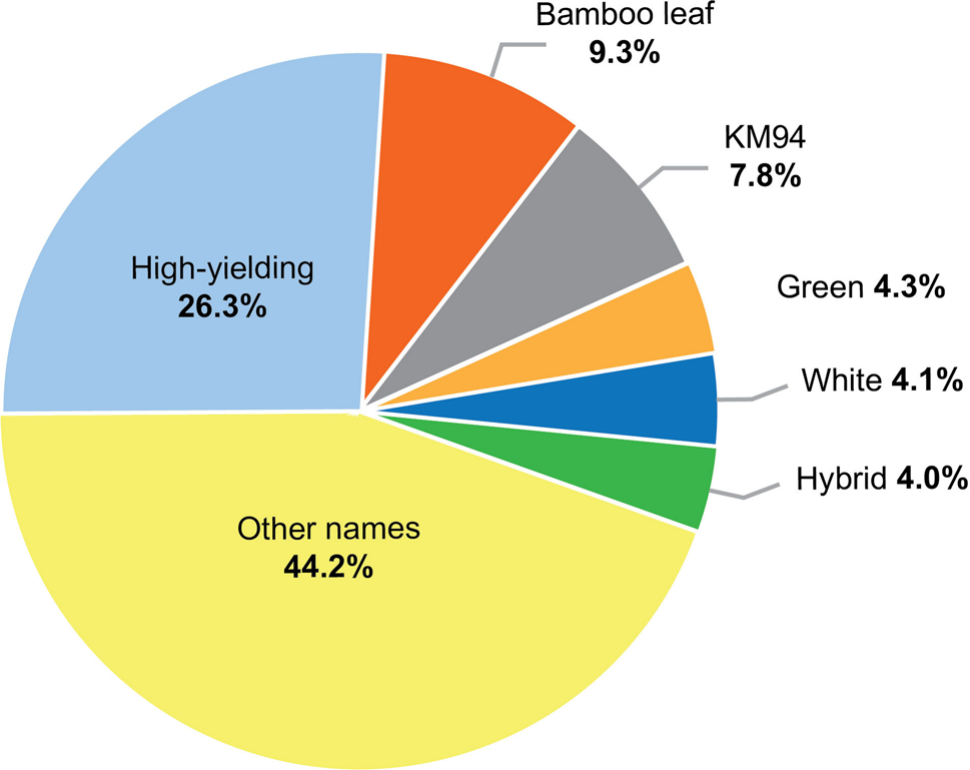
Numbers of varietal names recorded across 1570 genotypes collected in a survey of farms

Vietnamese farmers provided a range of names for cassava cultivar groups and specific varieties, and were knowledgeable of their geographic origins, institutional codes, and agro-morphological characteristics. Among these, high-yielding (26.3%), bamboo leaf (9.3%), KM94 (7.8%), green (4.3%), white (4.1%), and hybrid (4.0%) were the best known, and *high yielding* (or *Cao sản* in Vietnamese) was the most commonly recognized cultivar group (Fig. 3). Because this group has been improved genetically, its varieties were associated with the specific names such as KM94 (n = 122), KM140 (n = 33), Gon (n = 33), KM98 (n = 12), HL-S11 (n = 8), KM54 (n = 4), KM150 (n = 2), KM19 (n = 2), KM505 (n = 2), KM82 (n = 1), KM85 (n = 1), KM98-5 (n = 1), HL-S10 (n = 1), and SM937-26 (n = 1).

Geographic patterns of variety naming revealed that upland areas below 250 m above sea level (m a.s.l) had the highest diversity of names (82%). The remainder (12%) were found in mountain areas between 250 and 1000 m a.s.l. The South Eastern region had the highest diversity of nomenclature, with 35 different names, followed by the Central Highlands and North Central Coast regions, with 32 and 31 names, respectively. In the provinces Phú Yên, Bình Phước, Bình Thuận, and Tây Ninh, farmers used more than 12 variety names. But in the Tuyên Quang province, only one variety name was used by farmers (*KM94*).

### Diversity of SNPs

**Fig. 4:**
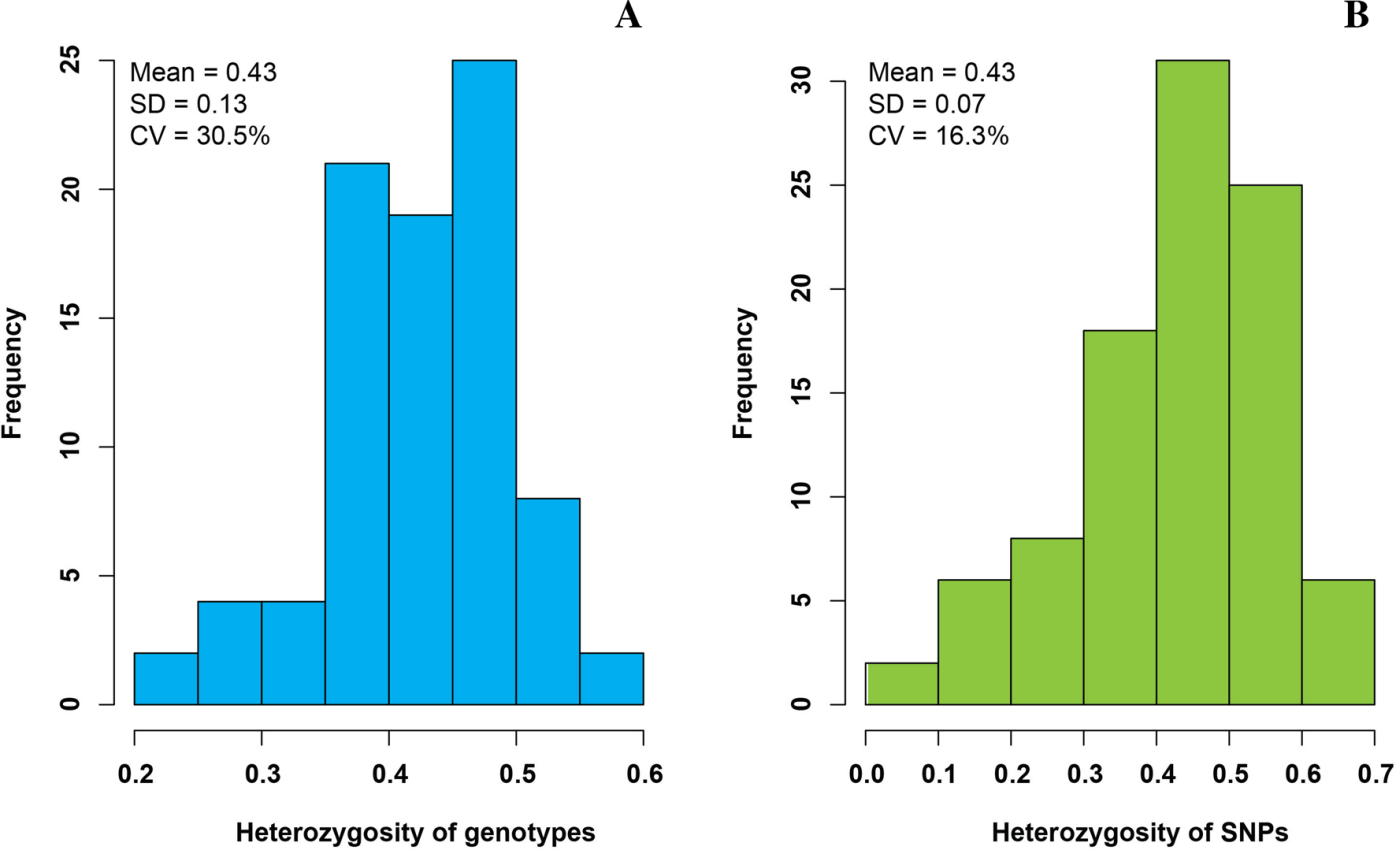
Frequency of heterozygosity observed across 96 SNPs markers selected (**a**) and from a set of 85 different cassava genetic groups identified across 1570 clones’ evaluated (**b**)

Approximately, 75% of the genotypes evaluated here showed observed heterozygosity above 0.35 (Fig. 4a). The observed heterozygosity ranged from 0.08 to 0.69 among 96 SNP markers selected using a SNPY-chip (Fig. 4b). The average observed heterozygosity (*Ho* = 0.43) was higher than expected (*He* = 0.40) across all 96 SNP markers, suggesting an excess of heterozygotes plants among the 1570 individuals evaluated, and among genotypes from the same agro-ecological zones (Table 1). This heterozygosity likely reflects the outcrossing nature (outbreeding) of cassava. An average of 0.32 PIC values were detected per SNP marker, ranging from 0.12 to 0.38, and 68% of these had PIC values over 0.30. A few SNP markers (SNPY-176, SNPY-179, and SNPY-181) had PIC values of less than 0.11, indicating intensive selection of these loci during domestication and/or breeding, or perhaps also due to genetic drift.

**Table 1:** Genetic diversity of cassava in six agro-ecological zones, as estimated by the single nucleotide polymorphism (SNP) analysis

Agoecologycal zone	Genotypes (n)	Farmer variety names (n)	% Duplicate genotypes	No. Heterozygous indivudials	*Ho*	*He*	*PIC*
North West	209	16	12.0	89	0.43	0.40	0.31
North East	256	18	16.0	112	0.44	0.40	0.31
North Central Coast	218	31	13.0	100	0.46	0.36	0.29
Central Highlands	419	32	24.5	180	0.43	0.36	0.29
South Central Coast	190	29	12.0	83	0.44	0.30	0.27
South East	278	35	17.0	121	0.44	0.38	0.30
Whole sample	1570	97	98.0	685	0.43	0.40	0.30

### Distribution of SNP variation richness

**Fig. 5:**
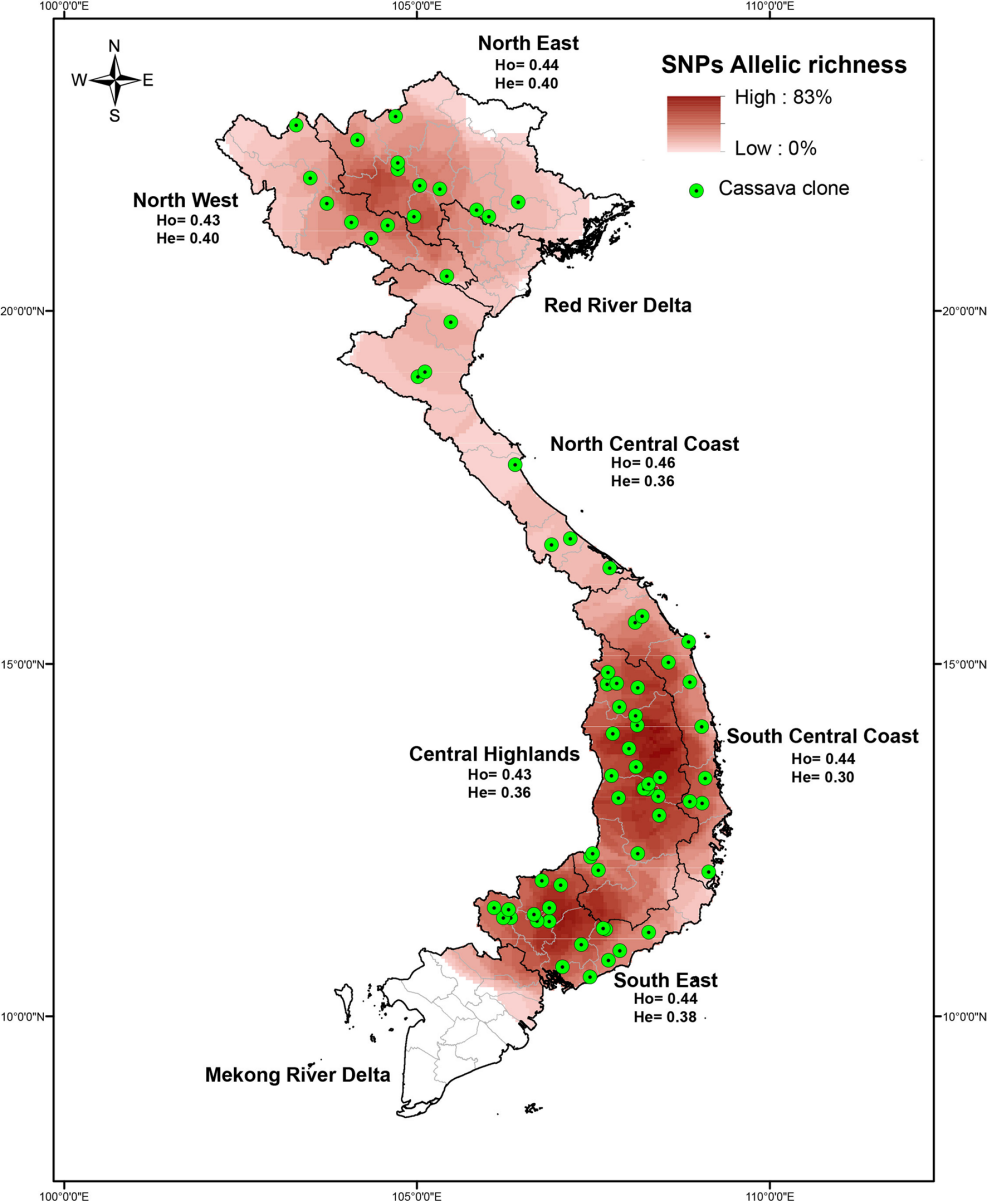
Spatial distribution of cassava plantations modeled based on allele richness; red areas indicate high concentrations of alleles with single nucleotide polymorphisms (SNPs; 63.73% to 82.94%)

Spatial distributions of allelic richness parameters for all loci in Vietnamese genotypes of cassava are presented in Fig. 5. Higher allele richness was clearly observed in Central Highland, South East, and North East zones, where more than 80% of alleles were identified. The Central Highland provinces Gia Lai and Đăk Lăk and the South Eastern provinces Bình Phước and Đồng Nai had the highest diversity, with up to 83% of allelic richness. These provinces are potential priority areas for on-farm conservation of varietal diversity or complementary collections for genebanks. A notably lower level of allelic richness was evident in North Central Coastal and North Westerns regions, although these zones had high percentages of different alleles.

### Genetic diversity of cassava genotypes among agro-ecological zones

Ninety-eight percent of genotypes had at least one duplicate among all samples, and 85 different cassava genetic groups were finally identified (Supplementary material 1). The highest percentage of duplicated genotypes was detected in the Central Highlands (24.5%), whereas duplication in North West and South Central Coast zones did not exceed 12% (Table 1). PIC values varied from 0.27 to 0.31 and averaged 0.30 for the total sample. The average observed heterozygosity (*Ho*) ranged from 0.43 to 0.46, and the North Central Coast zone had the highest level at 0.46 (Fig. 5). These diversity indexes (*He*) suggest that cassava samples from the North West and East zones have the highest genetic diversity (mean *He* = 0.40).

### Composition of cassava varieties

To identify varieties in farmer’s fields across 92 Vietnamese villages, we combined 85 different cassava genetic groups (Supplementary material Fig. 1) with a genotype reference set of 2000 unique improved landrace varieties from the Germplasm Banks of Latin American (CIAT, Colombia) and Asia (HLARC, RCRDC, AGI Vietnam).

**Fig. 6:**
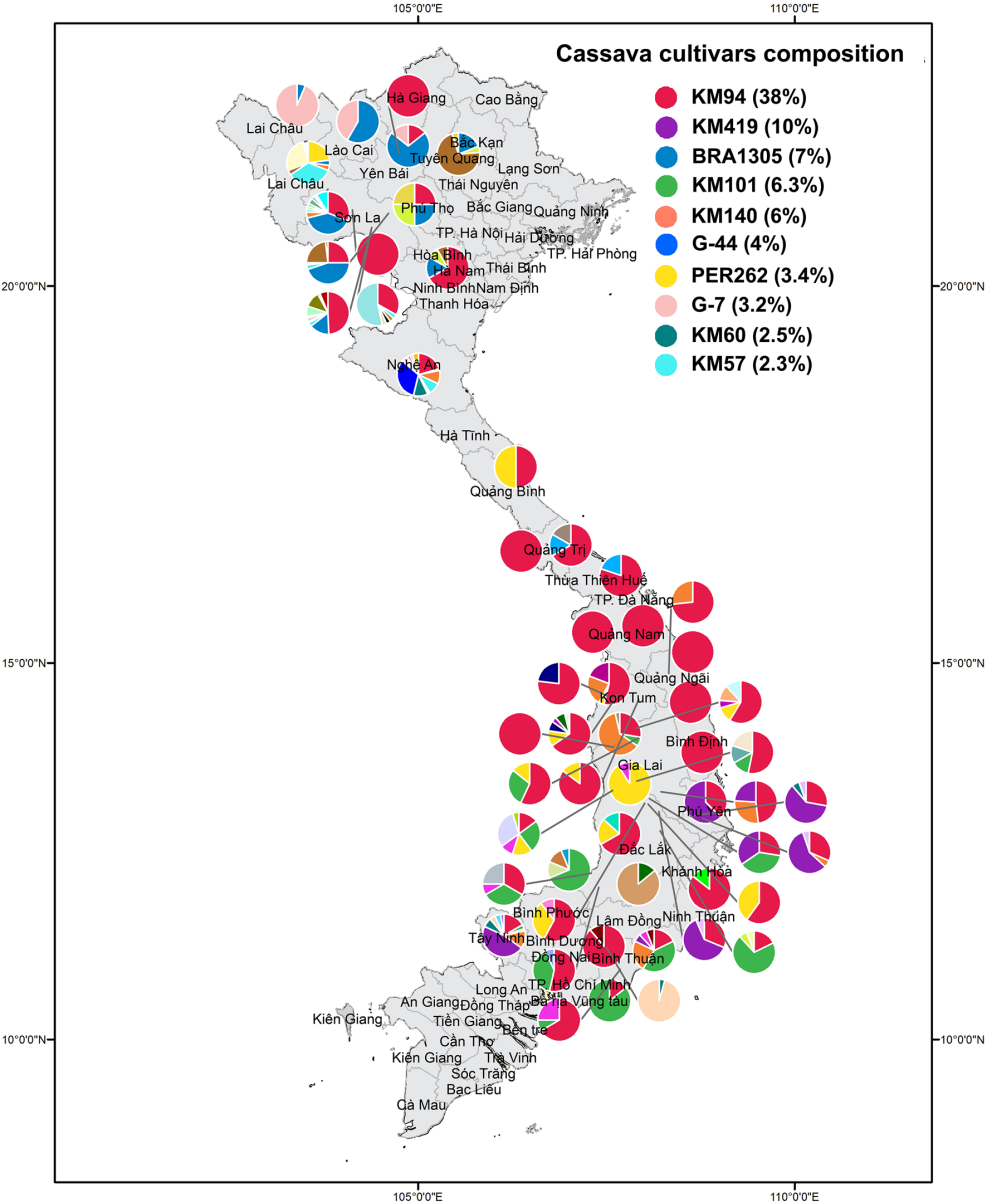
Spatial frequency distribution of the ten main cassava varieties identified in farmer’s fields across 92 villages in mainland Vietnam

Among varieties from Vietnamese farmer’s fields, KM94, KM419, BRA1305, KM101, KM140, PER262, KM60, KM57, and two unidentified varieties (G7 and G44) represented 82% of the frequency distribution (Fig. 6; Table 2 and Supplementary material Table 1). These enhanced varieties are the most common in Vietnam and the other 75 unique varieties (18%) are less frequently and evenly cultivated across the main cassava production zones in Vietnam. KM94 is the most popular variety and is known as Kasetsart 50, KU50, TAI16, or MKUC 28-77-3. This variety is very popular throughout Southeast Asia and was developed (Rayong 1 × Rayong 90) and originally released in Thailand in 1993. KM94 is grown by 38% of farmers across all agro-ecological zones between 0 and 1000 m.a.s.l. It is particularly common in the Central Highland provinces Đăk Lăk, Đăk Nông, Gia Lai, and Kon Tum. The second most popular variety is an official release from HLARC named KM419 (BKA900 × KM98-5) and is grown by 10% of the sampled farmers. KM419 is distributed from southern Vietnam to the Central Highlands and is particularly popular in Phú Yên and Tây Ninh provinces. Surprisingly, the 3rd and 4th most popular varieties (approximately 10%) are South American landraces. These are registered in the CIAT genebank as BRA1305 from Brazil and PER262 from Peru, but are referred to as KM98-5 and KM98, respectively, belong to genebank collections from Vietnam. The varieties KM101 and KM140 were grown by 6% of the present farmers and were particularly common in the Central Highland provinces Đăk Lăk, Đăk Nông, Gia Lai, and Kon Tum. KM60 and KM57 were introduced from Thailand and are present in only 2.5% and 2.4% of sampled Vietnamese farms, respectively, mainly in the North Central Coast (Fig. 6).

**Table 2:** Varieties most predominant in Vietnam and their Institutional or Genebank names in Asia and Colombia (CIAT)

Variety	Frequency (%)	Names given by farmers	No. samples	Status	Year of registration or release	Pedigree	Relationship first degree	Traits of interest
KM94	37.7	40	592	Improved	1995	Rayong 1 × Rayong 90	G44, Unique sample (2738)	High yield and starch content, highly branched
KM419	9.8	16	154	Improved	2016	BKA900 x KM318	IND59, Dòng 1	High yield
BRA1305	7.0	8	111	Landrace	Unknown	Unknown	Unique sample (346)	Suitable for fresh consumption
KM101	6.3	16	99	Improved	2017	Rayong 1 × CMR 23-149-128	Uniques samples (1864,397)	High yield and starch content
KM140	5.8	18	90	Improved	2007—2009	KM146 × KM98-1	Undetermined	High yield, low cyanogenic content, suitable for fresh consumption, best harvesting time
G44	4.0	11	64	Unknown	Unknown	Unknown	KM94	High yield and starch content
PER262	3.4	10	54	Landrace	Unknown	Unknown	KM57, Unique sample (473)	Suitable for fresh consumption
G7	3.2	6	51	Unknown	Unknown	Unknown	BRA1305, PER308	Suitable for fresh consumption
KM60	2.5	11	40	Improved	1995	Rayong 60 × SM937-26	Unique sample (2636), G49, COL1684	High yield, good root shape, early harvest, yellow flesh
KM57	2.3	9	37	Landrace	Unknown	Unknown	PER262	Suitable for fresh consumption

The CIAT’s SNPs reference library contains 2000 accessions of representative cassava landraces and improved varieties from Latin America and Asia. Herein, we identified additional genotypes that are not present in the collection. Among the ten most popular varieties in Vietnam, two lacked matches in the reference set. These varieties, G7 and G44, represented approximately 7% of cassava varieties in Vietnamese farmers’ fields. Both of these strains are restricted to well-defined regions. G44 is found only in the North Central Coast, where farmers refer to it as “high yielding,” suggesting that it is an improved variety. Similarly, G7 was found in the North of Vietnam and is known by farmers as “hybrid,” suggesting that it is the product of selection from breeding programs.

### Vietnamese cassava germplasm structure

**Fig. 7:**
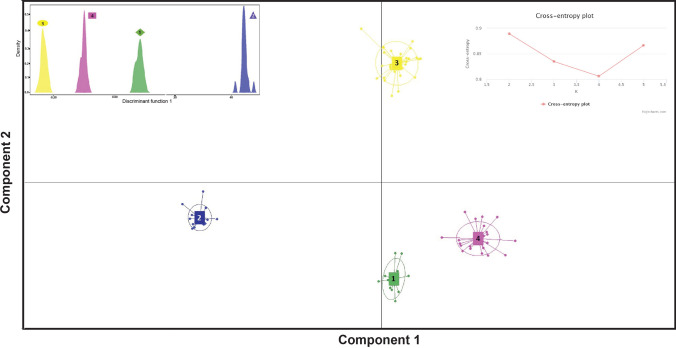
Scatterplots of discriminant analysis of principal components (DAPC) of a set of 85 varieties representing maximum variability

DAPC analyses were performed on 85 different varieties (G) representing the total genetic variability in Vietnam. DAPC analyses identify clear groups and subdivisions. The scatterplots in Fig. 7 clearly show four genetically distinct subgroups, and the ten most popular varieties in Vietnam are confidently distributed across these. Yet, no geographic pattern was found and the subgroup comprised a mix of varieties from different agro-ecological zones (Fig. 6). The largest subgroup (Cluster-I) is represented by 662 clones, predominantly of improved KM94 varieties (89%; G1) and an unidentified variety (9.6%; G44). The second subgroup (Cluster-II) comprised 307 samples and represented a mixture of a landrace from Latin America (BRA1305; 36%; G2), an improved variety (KM140; 30%; G6), and an unidentified variety (16.6%; G7). The Cluster-III comprises 168 samples and is represented by a landrace from Latin America (PER262; 32%; G21) and a potentially improved variety (KM57; 22%; G9). The last cluster subgroup (Cluster-IV) contains 400 clones that are represented by the nationally improved varieties KM419 (38.5%; G5) and KM101 (25%; G14).

### Genetic distances and cluster analysis

The average total genetic distance between genotypes of different agro-ecological zones was 0.025, with values ranging from 0.0050 to 0.046 (Table 3). The smallest distances were identified between samples from the North West and North East (0.005) and the North Central Coast and Central Highlands (0.009). These observations suggest poor differentiation or high prevalence of dominant cultivars, such as BRA1305 or KM140 and KM94, which are related by descent. Samples from the North West and North East differed most from South Central Coast samples, with genetic distances of > 0.044. We expect that these genetic distances follow different variety portfolios (e.g., Latin American landraces in the north) and contrasting environmental conditions.

**Table 3:** Genetic distances for all samples (n = 1570) by agro-ecological zone following Nei (1973)

Agro-ecological zone	North West	North East	North Central Coast	Central Highlands	South Central Coast
North East	0.0050				
North Central Coast	0.0242	0.0258			
Central Highlands	0.0200	0.0224	0.0086		
South Central Coast	0.0455	0.0445	0.0163	0.0152	
South East	0.0277	0.0287	0.0269	0.0218	0.0375

**Fig. 8:**
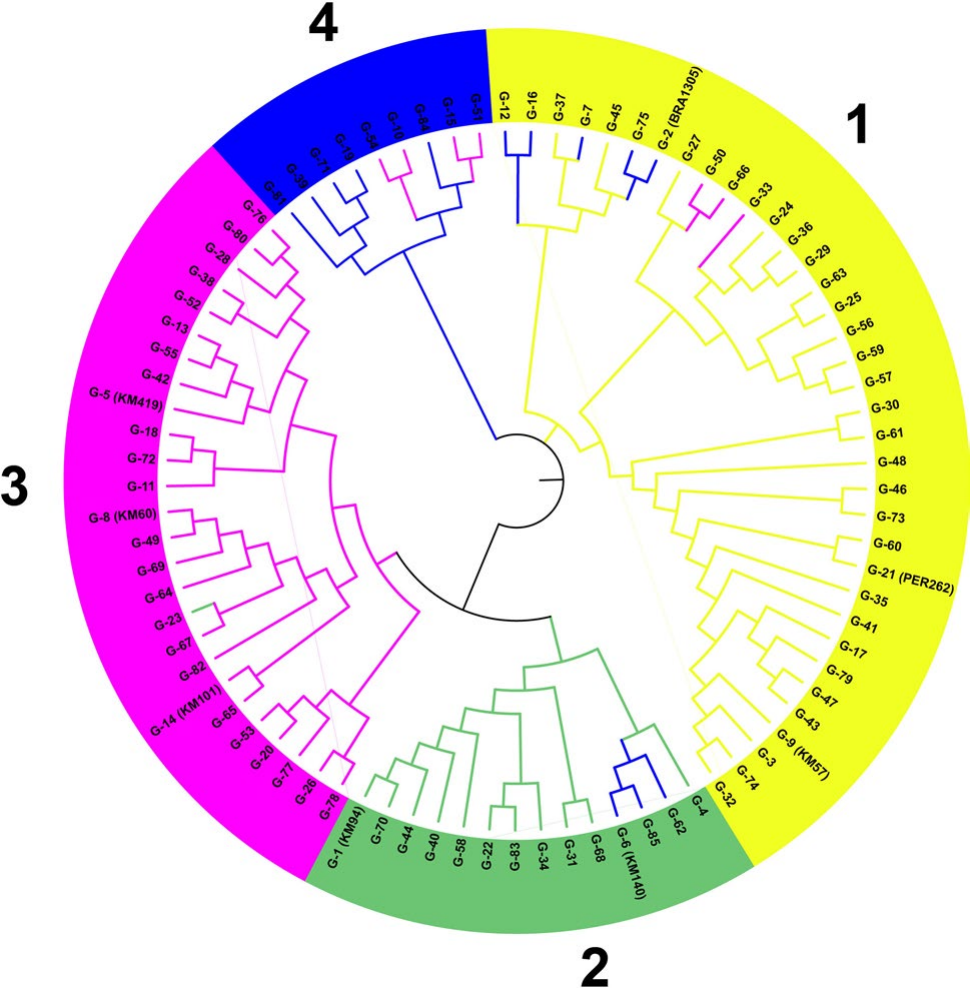
Cluster analysis of a portfolio of 85 varieties constructed with the neighbor joining (NJ) method using shared alleles to define genetic distances; differing internal colors represent DAPC groups

Genetic relationships between the present samples were identified in cluster analyses using the NJ method. The dendrogram in Fig. 8 is congruent with DAPC of the four main subgroups. Yet, a modest subpopulation structure was identified within each subgroup and high differentiation was observed between varieties of the same group. The first cluster (1) contains 14 varieties from four agro-ecological zones and includes three of the ten most popular varieties reported earlier (KM94, KM140, and G44). These three varieties are related to Rayong 1 by descent. The second cluster (2) includes nine genotypes that are present at low frequencies across Vietnam. The varieties of this cluster are not well represented in ex situ germplasm collections and were mostly from the North West and South Eastern agro-ecological zones. The third cluster (3) is the largest group, comprising 36 of the 85 distinct varieties distributed between three branches. This cluster contains the Latin American landraces BRA1305 and PER262 and hybrids (KM57 and G7) that were likely derived from these landraces. The fourth cluster (4) mainly includes varieties from the Central Highlands and the North Central Coast, and of the 26 genotypes, three are among the 10 most popular cassava varieties in Vietnam (KM60, KM101, and KM419). Collectively, these three varieties represent 19% of all samples collected from farmers’ fields. Similar to the first cluster, they are related to Rayong 1 by descent and to the original landraces introduced to Thailand by CIAT in the early 80s. Our NJ analyses and DAPC clearly indicate that two clusters of improved cassava varieties (1 & 4) are present in Vietnam. The other two clusters contain landraces and their derived hybrids (2 & 3).

### Unique cassava germplasm

SNP data from 1570 samples were analyzed in genetic duplication tests using NGSEP. These computations allowed identification of a set of 31 unique genotypes that were not duplicated with the other 1540 samples collected in Vietnam or with the 2000 genotypes of the CIAT cassava reference library (Table 4). This unique set represents only 2% of all 1570 collected and assessed varieties, but represents 35% of the 85 distinct varieties. These unique varieties are distributed across all four DAPC subgroups, which are supported by cluster analysis, and are grown by farmers from 18 villages in 13 provinces distributed across five of the six agro-ecological zones (0–903 m a.s.l). These varieties are also known by 18 distinct vernacular names, and are present at the highest concentrations (19 out of 31) in the Central Highland and North Central Coastal zones. This unique germplasm constitutes an important and unique genepool that should be added to genebank collections for use in future breeding programs.

**Table 4:**
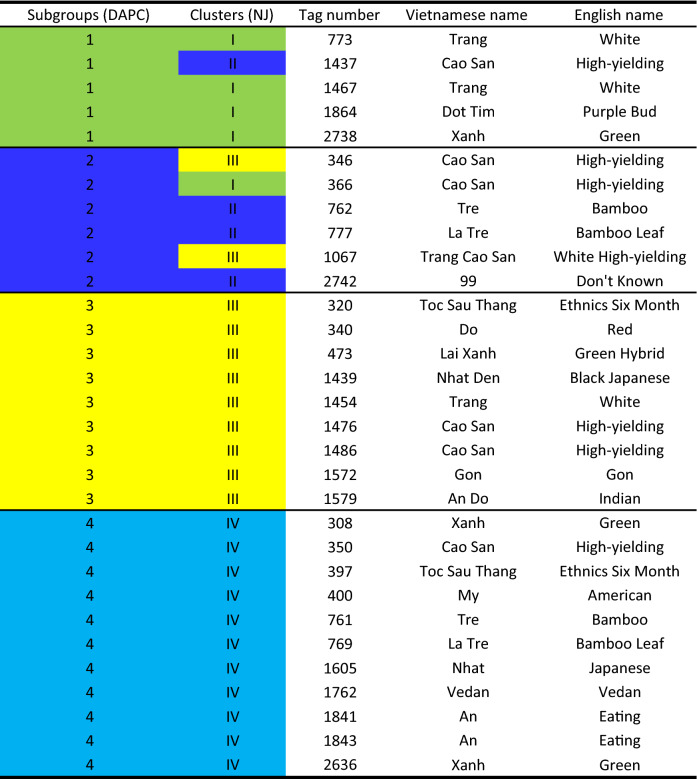
Set of 31 unique genotypes of cassava identified according to single nucleotide variants

Different colors are indicating four subgroups identified by DAPC and NJ analyses

## Discussion

### Farmer perceptions about cassava varieties

Smallholder farmers are important guardians of crop genetic diversity, even outside the center of origin (Altieri and Merrick 1987; Frison et al. 2011). Farming communities traditionally maintain knowledge of this genetic diversity through vernacular names for varieties. But depending on the context, informal naming of varieties can lead to overestimates or underestimates of crop diversity (Almekinders et al. 1994), because varieties can take different names between regions and communities (Lamprecht 2015). In our study, farmers named 97 different varieties from 1570 cassava genotypes that were sampled across six agro-ecological zones (Fig. 1). Lamprecht (2015) similarly found that cassava plants of the same clone took different names from different farmers, indicating that some farmers rename varieties after adoption and dissemination. Farmers distinguished different varieties mainly according to the morphology of vegetative parts, such as *Bamboo Leaf*, *Long Leaf*, *Purple Bud*, *Red Bud*, and *Red Branch*. Even though morphological characteristics offer limited resolution for varietal identification, extreme differences are still used by farmers to distinguish between varieties in their own localities (Lamprecht 2015). Another potential contributor of variety names is the exchange of plant materials among farmers in different zones (North, Central, and South), as reported by Balyejusa-Kizito (2007) in Uganda. In all provinces sampled, farmers most commonly (26.3%) referred to their varieties as high yielding, or *Cao sản* in Vietnamese. This name corresponds with old and modern improved varieties, such as KM94, KM101, and KM140. All of these improved varieties have been highly disseminated by cassava processing factories and government institutions in Vietnam (Kim et al. 2001, 2015). Our data also show that varietal breeder codes are difficult to adopt after release by national agricultural institutions, and agro-morphological characteristics such as high yielding are most commonly used by farmers to identify these strains. In general, farmers’ knowledge was highly variable and most farmers grew both local and improved varieties in the same plots. Hence, different farmers tended to plant different varieties.

### SNP diversity and genotyping

Ninety-six percent of the present SNP markers were effective for detecting genetic variability in the whole sample set (Table 1). The average PIC value was 0.32 across 1570 cassava samples and ranged from 0.07 to 0.38. Studies conducted by Kawuki et al. (2009), Oliveira et al. (2014), and Ferguson et al. (2012) showed lower PIC values (0.22–0.28) using a different set of SNPs markers in cassava germplasm from South America, Asia, and Africa). Hence, most of the loci markers used herein are highly informative. Moreover, the average observed heterozygosity (*Ho* = 0.43) was higher than expected (*He* = 0.40) in whole cassava samples and within each agro-ecological zone across all loci, suggesting high heterozygosity among the genotypes cultivated in Vietnam. Outcrossing (allogamous plants) and inbreeding-sensitive crops such as cassava are expected to have higher numbers of DNA polymorphisms (Kawuki et al. (2009), despite being clonally propagated. The present heterozygosity values are higher than those presented in the studies mentioned above (*Ho* < 0.36 and *He* < 0.38) for a wide geographic variety of germplasms. Similarly, Peña-Venegas et al. (2014) reported heterozygosity of *Ho* = 0.39 in a set of 173 cassava plants from the Colombian Amazon. Their set of plants comprised mainly landraces that were recognized locally according to traditional uses. Thus, our findings indicate higher heterozygosity of Vietnamese materials than reported by Peña-Venegas et al. (2014), who used the same set of SNP markers. These unexpected differences in *Ho* may reflect the geographic origins of the samples or the differing sizes and compositions of study sample sets (1570 vs. 173 plants). Despite the introduction and release of new varieties in Vietnam, proportions of LAC landraces were low in Vietnamese farmers’ fields, suggesting that these varieties contribute to the observed heterozygosity among cassava germplasm cultivated in Vietnam (Kim et al. 2015). Nguyen et al. (2013), Lamprecht (2015), and Nguyen et al. (2015a, b) reported high average observed heterozygosity (*Ho* = 0.67, 0.48, and 0.93, respectively) from studies using SSR markers, and these observations are concordant with our results. The high heterozygosity observed here and in previous studies was not corroborated by studies in Africa Fregene et al. 2003), Latin America, and the Caribbean (Siqueira et al. 2009; Montero et al. 2011), or with those from other South East Asian (SEA) countries (Thailand; Wangsomnuk et al. 2013; Fu et al. 2014). Hence, the genetic diversity of cassava germplasm in Vietnam is the result of anthropogenic selection and progress from the past few decades of breeding.

### Genotypic compositions of varieties

Correct identification of varieties is crucial for studies of the related impacts on-farm productivity and farmer incomes (Floro et al. 2017). In the present study, 917 household farms were surveyed and 1570 cassava stem samples were collected based on farmer’s knowledge of variety identities. The present cassava SNPY-array allowed identification and organization of these cassava samples into 85 genetic groups. These represent a rich and diverse collection when compared with the cassava reference variety set at the CIAT. We were also able to match genotype groups with varieties that have been conserved in the cassava Gene Banks of LAC and SEA, and we show that our Vietnamese set represents the major varieties KM94, KM419, BRA1305, KM101, KM140, PER262, KM60, KM57, and two unidentified varieties with high frequency of 82% (Table 1). Pedigree information for the most frequent variety KM94 (38%) was derived from hybridization of the Thai local varieties Rayong 1 and Rayong 90 (Kim et al. 2001). Kasetsart University in Thailand released this variety in 1992 under the name KU50. Yet it is listed in the global Gene Bank as TAI 16 (CIAT). KM94 is known for its high yield, high starch contents, and ability to adapt to unfavorable conditions (Kawano 2003). The second most popular variety KM419 (BKA900 × KM98-5) represented 10% of the frequency of distribution. It was proposed as a variety in 2006 by the Nong Lam University and Thai Nguyen University of Agriculture and Forestry, but was only officially released as a new variety in 2013. By 2015, KM419 was widely distributed in the main cassava production areas of Vietnam (Kim et al. 2015) and currently this variety represents 23% of the cultivated area in Vietnam (Le et al. 2019). The third most frequently distributed variety was the Latin American landrace BRA1305 (7%), also known as *Do Ha Tay* or *Bamboo Leaf*. This variety was exclusively found in the North of Vietnam below 750 m ASL. In addition, the variety KM101 (CMR29-56-101) was distributed across 6.3% of the country and was first used in 1989 as a high yielding variety in Vietnam. KM101 was officially released in 2015. This variety has been conserved at the CIAT genebank under the names TAI121 and TAI14, and according to pedigree and parental knowledge, was identified as CMR 29-56-101 (CMR 23-149-128 × Rayong1). KM140 showed a frequency distribution of only 6% and has been identified as a hybrid of KM98-1 and KM 36 by HLARC. This variety was released in 2017 to replace the main variety KM94 because its starch contents exceeded 26.2%. We also identified an important variety (G44) in Vietnamese farmers’ fields that was not found in any of the genebanks consulted. This unknown variety is closely related to KU50 and has a frequency distribution of 4% in the northern provinces of Lai Châu and Lào Cai. A second Latin American landrace from Peruvian origin, coded as PER262 at the CIAT genebank, had a 3.4% frequency distribution across North, Central and South of Vietnam. PER262 was identified as a duplicate of the strains CR63 and TAI9 (CIAT genebank accessions): this accession is showing high levels of disease resistance to cassava mosaic disease cause by the Sri Lanka Mosaic Virus (Becerra Lopez-Lavalle and Zhang personal Communication). Similarly, the last three most frequently found strains had first-degree relationships with a Latin American landrace that is present in the CIAT genebank. KM57 is known as VNM8 in the CIAT genebank and Xanh Vinh Phú in the HLARC genebank, and is related in first degree to PER262. This variety probably was grown in Vietnam prior to the breeding efforts by the CIAT and partners in Thailand, suggesting that it was grown at that time as a “farmer selected” material from PER262. The second unidentified variety among the top most frequently observed plants is related to the Peruvian landrace PER308 (CIAT genebank). Accordingly, KM60 (Rayong 60, Thailand) was closely related to the Colombian landrace COL1684, and was used in the first breeding efforts by the CIAT and partners in Thailand (Fig. 2).

These findings provide evidence that two improved cassava strains are grown today by Vietnamese farmers and account for 48% of the genotypes investigated in this study. These strains include a variety from Thailand KU50 (KM94) and a recently released variety from the national breeding program (KM419) Thus, cassava breeding in Vietnam has made remarkable progress since 1988, with prevalent adoption of locally selected genotypes since Vietnam began its cooperation with CIAT and the Asian Cassava Research Network (Fig. 2). For more than two decades, improved varieties have been distributed to a large number of households in Vietnam (Aye et al. 2015), and by 2013, these were grown on more than 93% of the total cassava area in Vietnam (Kim et al. 2015; Lamprecht 2015). Our results show that KM94 is the most dominant variety in Vietnamese farms, reflecting superior quality and productivity, as prioritized by the starch factories that promote its dissemination and use (Kim et al. 2015; Le et al. 2019); ; ; . Further studies of breeding and selection of cassava varieties that have high starch yields are required to enhance the adoption of high-yield varieties and to promote sustainable development. However, Le et al. (2019) suggested that KM94 had the highest adoption rate of 39% of the total area. KM19 also remains a dominant variety in Vietnam (23%), but is present at significantly lower numbers than expected from previous studies and expert opinion (Kim et al. 2015; Le et al. 2019).

The KM419 cassava production area was more than 50,000 ha in 2013, and its advantages over KM94 include 28% greater root yields (34.9–54.9 t/ha), starch contents (27.8%–30.7%), root dry matter contents (15.6–21.6 t/ha for 7–10 months after planting). This variety also has good root shape with white flesh, and is highly adaptable to various production conditions (Kim et al. 2015). Cassava breeding research in Vietnam has been strengthened and supported by the CIAT since 1988 (Malik et al. 2020), which has been evidenced in this study using molecular data The long-term investment in conservation and utilization of genetic resources for cassava in Latin America by the CIAT has led to increased genetic diversity of cassava varieties cultivated in developing countries such as Vietnam, and has facilitated access to key genetic information through DNA fingerprinting. This information helps to define the current composition of cassava germplasm and to understand the impact of these investments for small cassava growers.

### Genetic relationships among Vietnamese cassava crops

The average genetic distance between genotypes of different agro-ecological zones was 0.0247, indicating that the cassava gene pool in Vietnam is homogeneous and well distributed (Table 3). Cassava seeds were introduced into Vietnam in the middle of the eighteenth century, and the descendants of these seeds were used to establish nearly all of the commercial cassava farms in the country, and some in several Asian countries (Kawano 1978; Byrne 1984). The present DAPC and hierarchical NJ tree analyses both (Figs. 7, 8) indicated four main clusters. These assessments of genetic relationships between the present germplasm reveal significant diversity of cassava varieties in Vietnam. Moreover, most of the Vietnamese genotypes have similar genetic backgrounds and were likely derived from varieties that were introduced from Thailand since the 1990s (Kawano 2003; Kim et al. 2015; Ceballos and Hershey 2017), but also reflect gene flows due to movements of asexual seeds among farmers in different geographic origins. Sexual reproduction of cassava varieties offers an additional source of genetic variation, and seed plants (volunteer plants) have been observed in several farmers’ fields (Elias et al. 2001; Deputié et al. 2007; Peña-Venegas et al. 2014). Incorporation of cuttings from these plants into planting materials may also have contributed to the diversity of particular varieties. Accordingly, KM94 is predominantly present at a high frequency in farmers’ fields, but is closely related (1st degree relationship) to other varieties, such as G4, G18, G22, G28, G34, G40, and G44. Hence, these varieties may be derived from KM94 or from crosses with varieties that originated from KM94, as indicated by DAPC and NJ analyses.

### Conservation of Vietnamese cassava germplasm

A unique set of genotypes was present in 2% of the genotypes collected in this study, and these are cultivated across five agro-ecological zones containing 18 villages from 13 provinces (Table 4). This unique germplasm could have been generated from plants that reproduce clonally from spontaneous genotypes that reproduce sexually (volunteer seedlings; Deputié et al. 2010). Clones may have also accumulated fixed somatic mutations during vegetative propagation since the introduction of cassava to Asia in the 1800s (Kawano 1978; Lushai and Loxdale 2002). In vegetative propagated plants, such as cassava, these mutations can accumulate, resulting in differentiation of plants within the same clone (McKey et al. 2010). These 31 genotypes could be considered local Vietnam landraces that were selected over time, and the resulting potentially new varieties and their genetic diversities can therefore be considered a resource for breeders and farmers. Thus, the resulting increases in genetic diversity need to be protected with the help of conservation practitioners. In this study, we identified and characterized new genotypes that are making their way through the cassava agricultural envelope in Vietnam and are likely to dominate in a few years. Hence, conservation of this newly identified Vietnamese germplasm is warranted (Fig. 5). The genetic groups (85) identified using the SNPY-chip could be used to select accessions that conform with numerous ex situ germplasm collections of cassava. On-farm conservation of cassava appears the most efficient mechanism through which constant adaptations of germplasm can be achieved in Vietnam. These adaptations will limit the impact of climate change and biotic constraints on cassava production.

Nineteen of the 31 unique genotypes were concentrated in Central Highland (Kon Tum, Đăk Lăk, and Gia Lai) and North Central Coast zones (Nghệ An and Quảng Bình), which are priority areas for on-farm conservation programs. Similarly, it is necessary to include other areas that were identified in the spatial distributions, such as those with high allelic richness in the provinces of Gia Lai and Đăk Lăk (Central Highlands) and Bình Phước and Đồng Nai (South East; Fig. 5). These genotypes in these areas contribute an important genetic reservoir of unique individuals, and may carry genes that support adaptation to the environmental conditions of Vietnam. Ex situ conservation is certainly another strategy through which cassava germplasm can be preserved against extinguishing conditions, such as genetic erosion, climate change, and introductions of improved varieties. But only 25 of the 85 cassava genetic groups are currently conserved in ex situ Vietnamese genebanks, and two of the ten most important varieties grown in Vietnam are not present in these genebanks. Hence, on-farm conservation might greatly increase the diversity of Vietnamese accessions that are preserved in national (HLARC, RCRDC, and AGI) and international germplasm banks (CIAT).

## Future breeding implications

Vietnam has made great progress in adopting new breeding technologies and in propogating new cultivars in Asia. Determinations of genetic variability and investigations of its organization are essential for the establishment of interventions that promote more efficacious use of available genetic materials. Our analyses indicate considerable variability (*He* = 0.40) in the cultivated cassava materials that are currently present in Vietnam, and these were structured into four clusters using DAPC and NJ analyses (Figs. 7, 8). This genetic structure and variability information is of utmost importance for cassava breeders because it can be used to identify varieties that are more productive and resistant to specific phytosanitary problems, such as the current cassava mosaic disease (CMD) outbreak, which has limited cassava productivity in Asian countries (Wang et al. 2016; Uke 2018). In addition, loss of genetic diversity has been reported during selection programs and germplasm conservation procedures (Oliveira et al. 2014). Hence, it is necessary to complement pedigree and molecular marker information with morphological data for each variety. The resulting insights will allow the establishment of true relationships between phenotype and genotype variations (Ceballos et al. 2004, 2012). Plants with specific qualities that can be exploited for the production of food, biofuel, or starch can be considered sources of genotypes and should be used as the basis for superior genotype selections of more productive cultivars (Kawano 1978, 2003; Malik et al. 2020). These processes could start with selection of local populations with a focusing on direct farmer participation. Additionally, it is important to include individuals that carry unique genotypes to enrich the primary gene pool of cultivated genotypes. This strategy will conserve and improve the genetic resources of the species in Vietnam. These new genotypes should also provide good alternatives for the most adopted varieties (KM94 and KM419) in Vietnam.

The insights from our DAPC and NJ analyses will open new opportunities for the exploration of heterotic effects of the formed subgroups, and highlight progenitors with high genetic diversity that could be used in productive diallele crosses. Genetic differentiation between local and improved cassava strains suggests that local varieties harbor partly unique diversities that are potential genetic resources for breeding and are therefore needed to preserve production. Finally, this is the first study using SNP markers to examine genetic diversity in Vietnamese cassava crops. We demonstrate the effectiveness of this referenced technique for characterizing species germplasm and identifying duplicate accessions in genebanks (Floro et al. 2017; Singh et al. 2019).

## Conclusions

Our study illustrates the utility of SNP markers in genetic analyses of species with high levels of polymorphism, as is the case for cassava. Our analyses of genetic diversity and structure of cassava germplasm offer crucial information for the use of genetic resources in the search for commercially viable planting materials in Vietnam. In genetic diversity analyses, we detected higher variability of the Vietnamese gene pool than shown in previous studies using SNP markers. Specifically, the old improved variety KM94 (KU50) is distributed in all agro-ecological zones of Vietnam and was the dominating variety cultivated by farmers in this study (38%). We conclude that the genetic diversity in Vietnamese cassava farmers’ fields is only partially covered by CIAT accessions, suggesting that local varieties should be incorporated into germplasm banks of future collections.

## Supplementary Information

Below is the link to the electronic supplementary material.

Electronic supplementary material 1 (PDF 513 kb)

Electronic supplementary material 2 (PDF 171 kb)

## References

[CR1] Agency of Foreign Trade (2017) Production and export of cassava in 2017. (Ministry of Industry and Trade of the Socialist Republic of Vietnam). http://www.moit.gov.vn/en/News/508/production-and-export-of-cassava-in-2018.aspx. accessed 3 April 2017

[CR2] Allem AC (1994). The origin of *Manihot esculenta* Crantz (Euphorbiaceae). Genet Resour Crop Evol.

[CR3] Almekinders CJM, Louwaars NP, de Bruijn GH (1994). Local seed systems and their importance for an improved seed supply in developing countries. Euphytica.

[CR4] Altieri MA, Merrick LC (1987). In situ conservation of crop genetic resources through maintenance of traditional farming systems. Econ Bot.

[CR5] Aye TM, Fahrney K, Lefroy R (2015) “Research and Development in the Dynamic Cassava Sector of Southeast Asia”. In: Howeler R (ed) Cassava Production in Asia for Multiple Uses and for Multiple Markets. Proceedings of the Ninth Regional Cassava Workshop, held in Nanning, Guangxi, China P.R. 27 Nov – 3 Dec 2011. (CIAT, Hanoi, Vietnam). p 13–25. https://cgspace.cgiar.org/handle/10568/72642. accessed 4 April 2018

[CR6] Balyejusa-Kizito E, Chiwona-Karltun L, Egwang T, Fregene M, Westerbergh A (2007). Genetic diversity and variety composition of cassava on small-scale farms in Uganda: an interdisciplinary study using genetic markers and farmer interviews. Genetica.

[CR7] Becerra López-Lavalle LA, Clair H (2017). Molecular approaches in cassava breeding. Achieving sustainable cultivation of Cassava 2: genetics.

[CR8] Byrne D (1984). Breeding cassava. Plant Breed Rev.

[CR9] Carvalho RD, Guerra M (2002). Cytogenetics of *Manihot esculenta* Crantz (cassava) and eight related species. Hereditas.

[CR10] Ceballos H, Hershey CH, Campos H, Caligari P (2017). Cassava (*Manihot esculenta* Crantz). Genetic improvement of tropical crops.

[CR11] Ceballos H, Iglesias CA, Pérez JC, Dixon AGO (2004). Cassava breeding: opportunities and challenges. Plant Mol Biol.

[CR12] Ceballos H, Hershey C, Becerra-López-Lavalle LA, Janick J (2012). New approaches to cassava breeding. Plant breeding reviews 36.

[CR13] de Oliveira EJ, Ferreira CF, da Silva SV, de Jesus ON, Oliveira GA, da Silva MS (2014). Potential of SNP markers for the characterization of Brazilian cassava germplasm. Theor Appl Genet.

[CR14] Debouck D, Dominique D, Alexandra J, Hershey C, Llerme R (2011) Conservation of cassava genetic resource. https://cropgenebank.sgrp.cgiar.org/index.php/crops-mainmenu-367/cassava-mainmenu-232/conservation-mainmenu-213. accessed 7 June 2018

[CR15] Delaquis E, Andersen KF, Minato N, Cu TTL, Karssenberg ME, Sok S, Wyckhuys KAG, Newby JC, Burra DD, Srean P, Phirun I, Le ND, Pham NT, Garrett KA, Almekinders CJM, Struik PC, de Haan S (2018). Raising the stakes: cassava seed networks at multiple scales in Cambodia and Vietnam. Front Sustain Food Syst.

[CR16] Doyle J, Doyle J (1990). A rapid total DNA preparation procedure for fresh plant tissue. Focus.

[CR17] Duitama J, Quintero JC, Cruz DF, Quintero C, Hubmann G, Foulquié-Moreno MR, Verstrepen KJ, Thevelein JM, Tohme J (2014). An integrated framework for discovery and genotyping of genomic variants from high-throughput sequencing experiments. Nucleic Acids Res.

[CR18] Duputié A, David P, Debain C, McKey D (2007). Natural hybridization between a clonally propagated crop, cassava (*Manihot esculenta* Crantz) and a wild relative in French Guiana. Mol Ecol.

[CR19] Elias M, Penet L, Vindry P, Mckey D, Panaud O, Robert T (2001). Unmanaged sexual reproduction and the dynamics of genetic diversity of a vegetatively propagated crop plant, cassava (*Manihot esculenta* Crantz), in a traditional farming system. Mol Ecol.

[CR20] Ferguson ME, Hearne SJ, Close TJ, Wanamaker S, Moskal WA, Town CD, de Young J, Marri PR, Rabbi IY, de Villiers EP (2012). Identification, validation and high-throughput genotyping of transcribed gene SNPs in cassava. Theor Appl Genet.

[CR21] Fernández ME (2013). Comparison of the effectiveness of microsatellites and SNP panels for genetic identification, traceability and assessment of parentage in an inbred Angus herd. Genet Mol Biol.

[CR22] Floro VVO, Labarta RA, Becerra López-Lavalle LA, Martinez JM, Ovalle TM (2017). Household determinants of the adoption of improved cassava varieties using DNA fingerprinting to identify varieties in farmer fields: a case study in Colombia. J Agric Econ.

[CR23] Food and Agriculture Organization, FAO (2018) FAOSTAT Statistical Database. http://faostat.fao.org. accessed 29 Feb 2018

[CR24] Fregene MA, Suarez M, Mkumbira J, Kulembeka H, Ndedya E, Kulaya A, Mitchel S, Gullberg U, Rosling H, Dixon AG, Dean R, Kresovich S (2003). Simple sequence repeat marker diversity in cassava landraces: genetic diversity and differentiation in an asexually propagated crop. Theor Appl Genet.

[CR25] Frison EA, Cherfas J, Hodgkin T (2011). Agricultural biodiversity is essential for a sustainable improvement in food and nutrition security. Sustainability.

[CR26] Fu YB, Wangsomnuk PP, Ruttawat B (2014). Thai elite cassava genetic diversity was fortuitously conserved through farming with different sets of varieties. Conserv Genet.

[CR27] Ha CD, Quynh LTN, Hien NT, Thu PTL, Ham LH, Dung LT (2016). Morphological characterization and classification of cassava (*Manihot esculenta* Crantz) in Vietnam. Tap Chi Sinh Hoc.

[CR28] Hershey CH, Debouck D (2010) A global conservation strategy for cassava and wild *Manihot* species. A summary of stakeholder deliberations and recommendations prepared for the Global Crop Diversity Trust. https://www.croptrust.org/wp/wp-content/uploads/2014/12/cassava-strategy.pdf. accessed 26 April 2018

[CR29] Kawano K (1978). Genetic Improvement of Cassava (*Manihot esculenta* Crantz) for productivity. Trop Agric Res.

[CR30] Kawano K (2003). Thirty years of cassava breeding for productivity - biological and social factors for success. Crop Sci.

[CR31] Kawuki RS, Ferguson M, Labuschagne M, Herselman L, Kim DJ (2009). Identification, characterisation and application of single nucleotide polymorphisms for diversity assessment in cassava (*Manihot esculenta* Crantz). Mol Breed.

[CR32] Kim H, Bien PV, Quyen NT, Ngoan NT, Loan TP, Kawano K (2001) Cassava breeding and varietal dissemination in Vietnam from 1975 to 2000. In: Howeler R (ed) Cassava`s potential in Asia in the 21st Century: Present situation and future research and development needs. Proceedings of the sixth Regional workshop, held in Ho Chi Minh City, Vietnam, Feb. 21–25, 2000. S.L. Tan, Eds. (CIAT, Bangkok, Thailand). pp 147–160. http://ciat-library.ciat.cgiar.org/Articulos_Ciat/cassavas_potential_in_asia.pdf. accessed 2 September 2018

[CR33] Kim H, Mai NTT, Mai NB, Mai NB, Howeler R (2015) Cassava conservation and sustainable development in Vietnam. In: Howeler R (ed) Cassava production in Asia for multiple uses and for multiple markets. Proceedings of the Ninth Regional Cassava Workshop, held in Nanning, Guangxi, China P.R. 27 Nov – 3 Dec 2011. (CIAT, Hanoi, Vietnam). pp 35–56. https://cgspace.cgiar.org/handle/10568/72642. accessed 4 December 2018

[CR34] Lamprecht M (2015) Genetic Diversity and Farmers’ Selection of Cassava (*Manihot esculenta* Crantz) Varieties on Small-Scale Farms in Northern and Central Vietnam. Dissertation, Uppsala University

[CR35] Le DP, Labarta RA, de Haan S, Maredia M, Becerra LA, Nhu L, Ovalle T, Nguyen V, Pham N, Nguyen H, Nguyen H, Le K, Le HH (2019) Characterization of cassava production systems in Vietnam. Working Paper. CIAT Publication No. 480. International Center for Tropical Agriculture (CIAT). Hanoi, Vietnam. 54 p. https://hdl.handle.net/10568/103417. accessed 3 May 2019

[CR36] Lebot V, Atherton J, Rees A (2008). Cassava: postharvest quality and marketing. Tropical root and tuber crops: cassava, sweet potato, yams and aroids.

[CR37] Lushai G, Loxdale HD (2002). The biological improbability of a clone. Genet Res.

[CR38] Malik AI, Kongsil P, Nguyễn VA, Ou W, Sholihin SP, Sheela MN, Becerra LA, Utsumi Y, Lu C, Kittipadakul P, Nguyễn HH, Ceballos H, Nguyễn TH, Gomez MS, Aiemnaka P, Labarta R, Chen S, Amawan S, Sok S, Youabee L, Seki M, Tokunaga H, Wang W, Li K, Nguyễn HA, Nguyễn VD, Hàm LH, Ishitani M (2020). Cassava breeding and agronomy in Asia: 50 years of history and future directions. Breed Sci.

[CR39] Manichaikul A, Mychaleckyj JC, Daly RSS, Chen SM (2010). Robust relationship inference in genome-wide association studies. Bioinformatics.

[CR40] McKey D, Elias M, Pujol B, Duputié A (2010). The evolutionary ecology of clonally propagated domesticated plants. New Phytol.

[CR41] Montero-Rojas M, Correa AM, Siritunga D (2011). Molecular differentiation and diversity of cassava (*Manihot esculenta*) taken from 162 locations across Puerto Rico and assessed with microsatellite markers. AoB Plants.

[CR42] Nguyen HH, Dinh VC, Pham TN, Nguyen TN, Nguyen TH, Tran ML, Le L, Nguyen TV (2013). Study of genetic diversity in Vietnamese cassava varieties based on morphological and SSR analyses. J Agric Rural Dev.

[CR43] Nguyen PT, Nguyen CM, Pham MT, Tran ML, Le LQ, Chung LQ, Van NT (2015). Research on genetic diversity of cassava (*Manihot esculenta* Crantz) based on DNA polymorphism of GPSS1 gene. J Biol.

[CR44] Nguyen PT, Nguyen CM, Phan MT, Nguyen HH, Tran ML, Lien LQ, Trung LQ, Binh LT, Van NT (2015). Research on genetic diversity of cassava (*Manihot esculenta* Crantz) based on SSR markers. J Agric Rural Dev.

[CR45] Olsen KM, Schaal BA (1999). Evidence on the origin of cassava: phylogeography of *Manihot esculenta*. Proc Natl Acad Sci USA.

[CR46] Padi FK, Ofori A, Takrama J, Djan E, Opoku SY, Dadzie AM, Bhattacharjee R, Motamayor JC, Zhang D (2015). The impact of SNP fingerprinting and parentage analysis on the effectiveness of variety recommendations in cacao. Tree Genet Genomes.

[CR47] Peña-Venegas C, Stomph T, Verschoor G, Lopez-Lavalle LA, Struik P (2014). Differences in manioc diversity among five ethnic groups of the Colombian Amazon. Diversity.

[CR48] Rafalski A (2002). Applications of single nucleotide polymorphisms in crop genetics. Curr Opin Plant Biol.

[CR49] Ramos Abril LN, Pineda LM, Wasek I, Wedzony M, Ceballos H (2019). Reproductive biology in cassava: stigma receptivity and pollen tube growth. Commun Integr Biol.

[CR50] Singh N, Wu S, Raupp WJ, Sehgal S, Arora S, Tiwari V, Vikram P, Singh S, Chhuneja P, Gill BS, Poland J (2019). Efficient curation of genebanks using next generation sequencing reveals substantial duplication of germplasm accessions. Sci Rep.

[CR51] Siqueira MV, Queiroz-Silva JR, Bressan EA, Borges A, Pereira KJ, Pinto JG, Veasey EA (2009). Genetic characterization of cassava (*Manihot esculenta*) landraces in Brazil assessed with simple sequence repeats. Genet Mol Biol.

[CR52] Uke A, Hoat TX, Quan MV, Liem NV, Ugaki M, Natsuaki KT (2018). First report of Sri Lankan cassava mosaic virus infecting cassava in Vietnam. Plant Dis.

[CR53] van Zonneveld M, Scheldeman X, Escribano P, Viruel MA, Van Damme P, Warcia W, Tapia C, Romero J, Sigueñas M, Hormaza JI (2012). Mapping genetic diversity of cherimoya (*Annona cherimola* Mill.): application of spatial analysis for conservation and use of plant genetic resources. PLoS ONE.

[CR54] Visser B, Engels JMM, Rao VR, Dempewolf J, MvD W, Hawtin G, McGuire P (2010). The state of diversity. The Second Report on the State of the World’s Plant Genetic Resources for Food and Agriculture.

[CR55] Wang W, Feng B, Xiao J (2014). Cassava genome from a wild ancestor to cultivated varieties. Nat Commun.

[CR56] Wang HL, Cui XY, Wang X, Liu SS, Zhang ZH, Zhou XP (2016). First report of Sri Lankan cassava mosaic virus infecting cassava in Cambodia. Plant Dis.

[CR57] Wangsomnuk PP, Ruttawat B, Wongtiem P (2013). Identification of genetically distinct cassava clones from on-farm plantations to widen the Thai cassava breeding gene pool. Am J Plant Sci.

[CR58] Wossen A, Tessema G, Abdoulaye T, Rabbi I, Olanrewaju AS, Bentley J, Alene A, Feleke S, Kulakow PA, Asumugha GN, Abass A, Toluka M, Manyong, VM (2017) The cassava monitoring survey in Nigeria: final report. Ibadan: IITA. 66 p. https://cgspace.cgiar.org/handle/10568/80706. accessed 10 October 2020

[CR59] Wossen A, Alene A, Abdoulaye T, Feleke S, Rabbi IY, Manyong V (2018). Poverty reduction effects of agricultural technology adoption: the case of improved cassava varieties in Nigeria. J Agric Econ.

[CR60] Zhou L, Matsumoto T, Tan H-W, Meinhardt LW, Mischke S, Wang B, Zhang D (2015). Developing single nucleotide polymorphism markers for the identification of pineapple (*Ananas comosus*) germplasm. Hortic Res.

