# Supplementary material for: DNA fingerprinting reveals varietal composition of Vietnamese cassava germplasm (Manihot esculenta Crantz) from farmers’ field and genebank collections
Source: Plant Mol Biol. 2021 Feb 25;109(3):215–32. doi: 10.1007/s11103-021-01124-0 (PMC9162981; doi:10.1007/s11103-021-01124-0)
Supplement: Supplementary file 1 — Electronic supplementary material 1 (PDF 513 kb) [file 11103_2021_1124_MOESM1_ESM.pdf]

Electronic Supplementary Material Information for

DNA fingerprinting reveals varietal composition of Vietnamese cassava germplasm (*Manihot esculenta* Crantz) from farmers' field and genebank collections.

John Ocampo, Tatiana Ovalle, Ricardo Labarta, Dung Phuong Le, Stefan de Haan, Nguyen Anh Vu, Le Quy Kha, Luis A. Becerra Lopez-Lavalle*

E-mail: l.e.becerra@cgiar.org

This PDF file includes:

Supplementary text Fig. 1

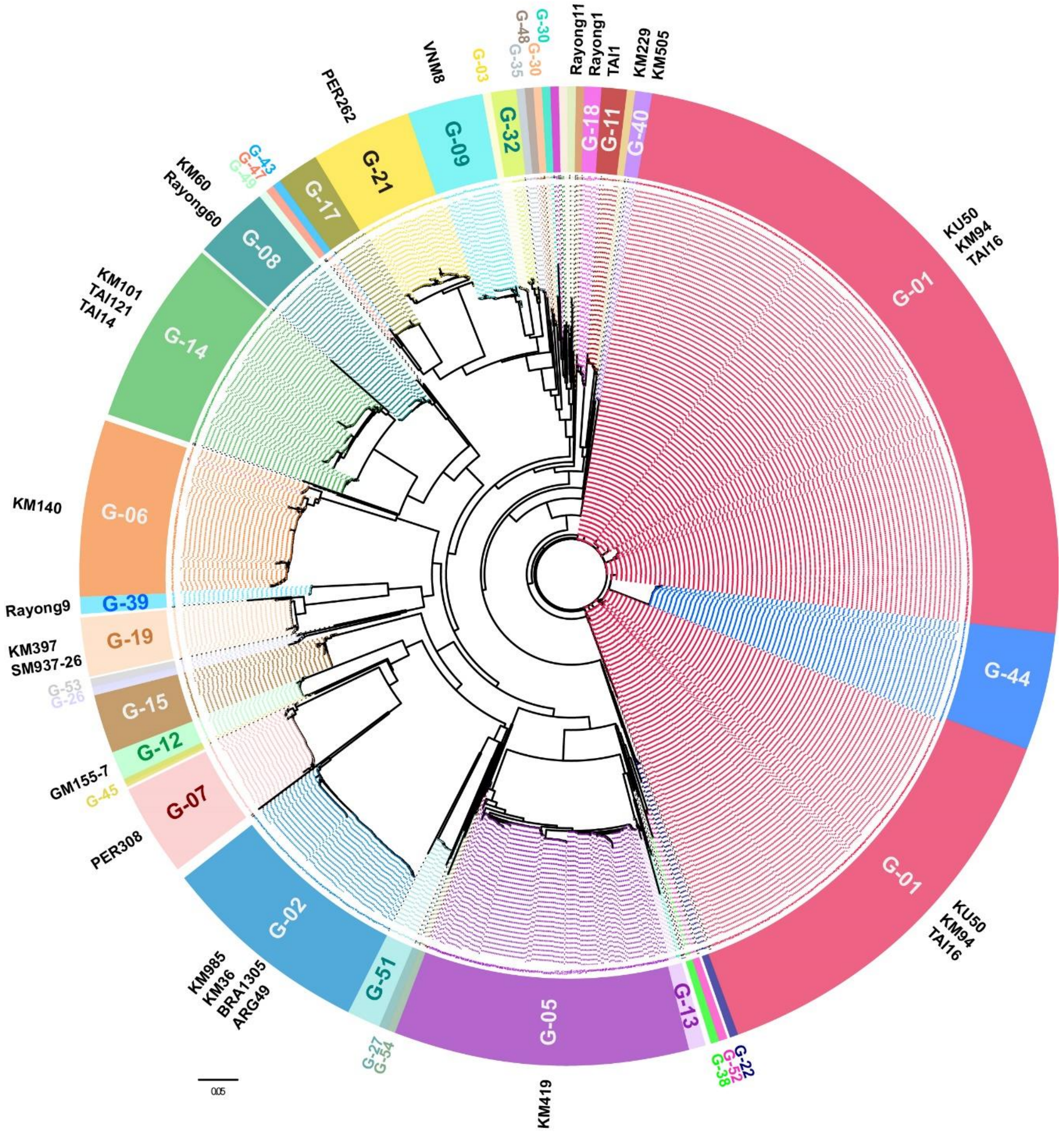

Fig. 1 Cluster analysis genetic diversity of cassava clones using the NJ method. A total 85 different cassava genetic groups identified across 1570 clones evaluated.
