# Supplementary material for: DNA fingerprinting reveals varietal composition of Vietnamese cassava germplasm (Manihot esculenta Crantz) from farmers’ field and genebank collections
Source: Plant Mol Biol. 2021 Feb 25;109(3):215–32. doi: 10.1007/s11103-021-01124-0 (PMC9162981; doi:10.1007/s11103-021-01124-0)
Supplement: Supplementary file 2 — Electronic supplementary material 2 (PDF 171 kb) [file 11103_2021_1124_MOESM2_ESM.pdf]

Electronic Supplementary Material Information for

DNA fingerprinting reveals varietal composition of Vietnamese cassava germplasm (*Manihot esculenta* Crantz) from farmers' field and genebank collections.

John Ocampo, Tatiana Ovalle, Ricardo Labarta, Dung Phuong Le, Stefan de Haan, Nguyen Anh Vu, Le Quy Kha, Luis A. Becerra Lopez-Lavalle*

E-mail: l.e.becerra@cgiar.org

This PDF file includes:

Supplementary text Table. 1

Table 1. Frequency of the most predominant varieties in Vietnam and their Institutional or Genebank names in Asia and Colombia (CIAT).

Variety	No. Samples	Frequency (%)	Institutional or Genebank names	Genbanks-Institutions
KM94	592	37.7	KU50, TAI16, MKUC 28-77-3, Kasetsart 50	Kasetsart University (KU)
KM419	154	9.8	D3	Hung Loc Agricultural Research Center (HLARC)
BRA1305	111	7	KM36, KM981, KM985, 11Sa12Q, DT03, HL2004-28, HM 125, KM140Q, KM21-10, KM225, KM419Q, KM94C, KM94Q, KM987Q, LC2, NA1, SC205, SM 937.26.11, Sa06, TMS60444, Do Ha Tay	International Center for Tropical Agriculture (CIAT), Hung Loc Agricultural Research Center (HLARC), Root Crop Research and Development Center (RCRDC), AGI.
KM101	99	6.3	TAI121, TAI14	Kasetsart University (KU)
KM140	90	5.8	Dòng 6, KM104.3, KM140-3, KM227, KM298-1, KM98-5-10, RAYONG 2	Hung Loc Agricultural Research Center (HLARC)
G44	64	4		
PER262	54	3.4	KM48, KM49, KM63-1, KM79, KM98-5, Gon, KM140, KM294-2, KM299-3, KM310-1, KM318-7,CR30, CR63, TAI9,IND103, IND108, IND115, IND117, IND120, IND131, IND177, IND180, IND215, IND222, IND252, IND61, IND75, IND76, IND94, IND96, TAI9	International Center for Tropical Agriculture (CIAT), Hung Loc Agricultural Research Center (HLARC), Root Crop Research and Development Center (RCRDC), AGI.
G7	51	3.2		
KM60	40	2.5	TAI8	International Center for Tropical Agriculture (CIAT), Hung Loc Agricultural Research Center (HLARC)
KM57	37	2.3	VNM8, Xanh Vinh Phú	International Center for Tropical Agriculture (CIAT), Hung Loc Agricultural Research Center (HLARC)
